# Author Correction: Physiological response of mammary glands to *Escherichia coli* infection:A conflict between glucose need for milk production and immune response

**DOI:** 10.1038/s41598-021-84933-z

**Published:** 2021-03-02

**Authors:** Shlomo E. Blum, Dan E. Heller, Shamay Jacoby, Oleg Krifuks, Uzi Merin, Nissim Silanikove, Yaniv Lavon, Nir Edery, Gabriel Leitner

**Affiliations:** 1 National Mastitis Reference Center, Kimron Veterinary Institute, Ministry of Agriculture and Rural Development, P.O. Box 6, 50250 Bet Dagan, Israel; 2 Department of Animal Sciences, Robert H. Smith Faculty of Agriculture, Food and Environment, The Hebrew University of Jerusalem, P.O. Box 12, 76100 Rehovot, Israel; 3 Institute of Animal Science, A.R.O. The Volcani Center, P.O. Box 6, 50250 Bet Dagan, Israel; 4 Food Quality and Safety, Postharvest and Food Sciences, A.R.O. The Volcani Center, P.O. Box 6, 50250 Bet Dagan, Israel; 5 Israel Cattle Breeders Association, 38900 Caesarea, Israel

Correction to: *Scientific Reports* 10.1038/s41598-020-66612-7, published online 15 June 2020

Nir Edery was omitted from the author list in the original version of this Article. This has been corrected in the PDF and HTML versions of the Article.

The Author Contributions statement now reads:

“S.B., D.H. and G.L. were responsible for performing the experiments, collecting data, experimental design, data analysis and preparing the manuscript draft; S.J. was responsible for animal husbandry and care; O.K. performed the laboratory work; Y.L. performed the statistical analyses; N.E. performed histological exams, provided histological pictures and descriptions; U.M., N.S. S.B. and G.L. analyzed the data and helped in writing and editing the manuscript. All authors read and approved the final manuscript.”

